# A triglyceride‐rich lipoprotein environment exacerbates renal injury in the accelerated nephrotoxic nephritis model

**DOI:** 10.1111/cei.13111

**Published:** 2018-03-09

**Authors:** M. F. Saja, H. T. Cook, M. M. Ruseva, M. Szajna, M. C. Pickering, K. J. Woollard, M. Botto

**Affiliations:** ^1^ Department of Medicine Imperial College London London UK

**Keywords:** dyslipidaemia, hypertriglyceridaemia, macrophage, renal injury

## Abstract

Hyperlipidaemia accompanies chronic renal disease either as a consequence of the renal dysfunction or as part of generalized metabolic derangements. Under both situations, the lipid profile is characterized by accumulation of triglyceride‐rich lipoproteins (TGRLs). This lipid profile is recognized as a risk factor for cardiovascular complications. Whether it may pose a risk for renal injury as well remains unclear. A hyper‐TGRL state was generated in C57BL/6 mice using poloxamer‐407 (P‐407) and immune complex‐mediated renal injury was triggered using the accelerated nephrotoxic nephritis (ANTN) model. The hyper‐TGRL animals were hypersensitive to ANTN demonstrated by greater haematuria and glomerular cellularity. These changes were accompanied by increased glomerular accumulation of CD68^+^ macrophages. The hypersensitive response to ANTN was not seen in low‐density lipoprotein receptor knock‐out mice fed with a high fat diet, where triglyceride levels were lower but cholesterol levels comparable to those obtained using P‐407. These data indicate that a hyper‐TGRL state might be more detrimental to the kidneys than low‐density lipoprotein‐driven hypercholesterolaemia during immune complex‐mediated nephritis. We speculate that the hyper‐TGRL environment primes the kidney to exacerbated renal damage following an inflammatory insult with increased accumulation of macrophages that may play a key role in mediating the injurious effects.

## Introduction

The detrimental effects of abnormal lipid and lipoprotein profiles on the kidney are well documented 1. Dyslipidaemia frequently complicates the course of renal diseases and contributes to their progression 1, 2. Dyslipidaemia may develop as part of a more global metabolic derangement, as occurs in type 2 diabetes (T2D) and the metabolic syndrome (MS), or as a consequence of an immune‐mediated inflammatory state, as seen in patients with systemic lupus erythematosus (SLE), where its occurrence is known to increase the risk of renal complications 2, 3, 4, 5. Whether or not dyslipidaemia complicates or precedes the course of renal disease, disturbances in lipid and lipoprotein metabolism are thought to promote, if not incite, renal injury 6.

A variety of animal studies demonstrate accelerated renal injury upon high fat (HF) feeding 7, 8, 9. The lupus‐prone New Zealand black and white mice [(NZB×W)F_1_] develop worse renal disease and increased mortality on a HF diet 7. Guinea pigs fed a high cholesterol (CHOL) diet exhibited enhanced glomerular cellularity with mesangial matrix expansion 8, while rats fed a high CHOL diet develop significant proteinuria 9. These studies have focused primarily on the effects of changes in CHOL levels on experimental renal disease, but the lipid profile in metabolic diseases such as T2D and MS is characterized by hypertriglyceridaemia secondary to accumulation of triglyceride‐rich lipoproteins (TGRLs), i.e. very low‐density lipoproteins (VLDL) 10, 11. This hyper‐TGRL lipid profile also occurs in other non‐metabolic inflammatory conditions such as systemic lupus erythematosus (SLE), where it is associated with enhanced morbidity and mortality 5, 12. Triglycerides (TGs) constitute the majority of dietary fat and there is an influx of TGRLs following each meal 13; however, the role of hypertriglyceridaemia and a hyper‐TGRL state in the pathogenesis of renal disease remain poorly investigated 8. This is because inducing isolated hypertriglyceridaemia in laboratory animals is extremely difficult. Dietary induction of hypertriglyceridaemia in animals on high sugar diets is associated with hyperglycaemia, itself a risk factor for renal disease 14. Genetically modified hypertriglyceridaemic mice display complex metabolic derangements, making it very difficult to distinguish the contribution of TGs to the general metabolic picture 15, 16. To determine the effect of TGRLs on experimental renal injury we adopted the poloxamer‐407 (P‐407)‐induced model of hyperlipidaemia 17, 18. P‐407 is a surfactant that induces dose‐dependent hyperlipidaemia in rodents through inhibition of lipoprotein lipase (LpL) 18. The hyper‐TGRL state is characterized by the accumulation of VLDL leading to gross hypertriglyceridaemia accompanied by low HDL levels, a lipid profile comparable to that observed in T2D, MS and SLE 5, 10. We have shown 19 that the P‐407‐induced hyper‐TGRL environment alters the distribution of circulating monocyte subsets, specifically causing a drop in Gr1^low^ monocyte numbers 20, 21. This was associated with extensive accumulation of CD68^+^ macrophages within tissues, including the kidneys. We interpreted these changes as the result of the promotion of Gr1^low^ monocyte migration from blood to surrounding tissues driven by the hyper‐TGRL environment 19. Unexpectedly, the accumulation of CD68^+^ macrophages in the kidneys during hyper‐TGRL conditions did not result in renal damage 19. However, we hypothesized that the presence of increased macrophage numbers in the kidneys under hyper‐TGRL conditions would result in exacerbated renal damage following an inflammatory trigger. We tested this hypothesis by subjecting C57BL/6 (B6) mice to accelerated nephrotoxic nephritis (ANTN) during P‐407‐induced hyper‐TGRL.

## Materials and methods

### Mice

C57BL/6 (B6) mice were purchased from Charles River Laboratories (Wilmington, MA, USA). C57BL/6.LDLR‐deficient mice (*Ldlr^–/–^*) were purchased from Jackson Laboratory (Bar Harbor, ME, USA). Mice aged 8–12 weeks (weighing between 18 and 20 g) were used for all experiments. The HF diet used consisted of a cholate‐free western‐type HF diet with the following composition: 15% cocoa butter, 1% corn oil, 0·25% cholesterol, 40·5% sucrose, 10% cornstarch, 20% casein, total fat content 16% (Arieblok Diet W, cat. 4021.06) purchased from (Hope Farms, Woerden, the Netherlands). All animals were housed in individually ventilated cages. All procedures were carried out according to the Institutional guidelines and the use of experimental animals was performed following the ARRIVE (Animal Research: Reporting of In Vivo Experiments) guidelines. Animal studies were approved by the UK Home Office.

### P‐407 administration and lipid levels

P‐407 (Pluronic F‐127, Cat. no. P2443; Sigma‐Aldrich, Dorset, UK) was dissolved overnight in cooled sterile phosphate‐buffered saline (PBS). Mice were injected intraperitoneally (i.p.) with 200 μl of P‐407 solution (10, 5 or 2·5 mg equivalent to 0·5, 0·25 and 0·125 g/kg, respectively) or PBS every other day. According to the experimental design blood samples were collected into heparin tubes at different time‐points. In P‐407‐treated animals, blood collection was performed 24 h following drug administration. CHOL and TG levels were measured by colorimetric assay using the cholesterol and triglyceride infinity reagent (TR13421 and TR22421, respectively; Thermo‐Scientific, Middletown, VA, USA).

### Accelerated nephrotoxic nephritis (ANTN)

B6 mice were treated with P‐407 injections for 9 days and then immunized i.p. with 200 μg sheep immunoglobulin IgG (I‐5131; Sigma) in complete Freund's adjuvant (CFA; Sigma). Five days later, 200 µl 10% sheep nephrotoxic serum (NTS) was administered intravenously (i.v.) 22. During the course of the experiment proteinuria and haematuria levels were determined using Hema‐Combistix (Bayer, Reading, UK). Blood was collected prior to P‐407 injection, before NTS administration and at the end of the experiment. Serum urea was measured using an ultraviolet (UV) method kit (R‐Biopharm Rhone, Flasgow, UK), according to the manufacturer's instructions. Thirteen days after the NTS injection, overnight urine collection was performed and the animals were then killed. Paraffin sections were stained with period acid‐Schiff (PAS) for glomerular cellularity scoring. PAS‐stained kidney sections were scored blindly, as described previously 22.

### Injection of nephrotoxic serum

B6 mice were treated with either 10 mg P‐407 or PBS i.p. for 2 weeks and then allocated randomly to either receiving 200 µl of 10% NTS intravenously or not (*n* = 5/group). Urine samples were collected 24 h after the NTS administration and proteinuria measured using the sulphosalicylic acid method 23.

### Immunohistochemistry

CD68 staining was performed on periodate–lysine–paraformaldehyde (PLP)‐fixed kidney tissue followed by 7% sucrose. Acetone‐fixed 5 μm‐thick sections were then blocked sequentially with 10% milk and 0·03% hydrogen peroxide. The sections were stained with anti‐CD68 (FA‐11, MCA 1957; AbD Serotec, Kidlington, UK) and developed using the Polink‐2 plus HRP detection kit (no. D46‐18; GBI Laboratories, Bothwell, WA, USA). Analysis of glomerular CD68 staining was performed using a Zeiss microscope. Quantification was carried out as described previously 24.

For mouse C3 and IgG glomerular staining, snap‐frozen kidney tissues were cut at a thickness of 5 μm and fixed in acetone. Sections were then blocked with 20% normal goat serum and stained with either fluorescein isothiocyanate (FITC)‐labelled polyclonal goat anti‐mouse C3 (MP Biomedical, Cambridge, UK) or FITC‐conjugated polyclonal goat anti‐mouse IgG Fcγ chain‐specific antibody (Sigma‐Aldrich). Sections were then washed and mounted using Vectashield Hard Set mounting medium (Vector Laboratories, Peterborough, UK). Murine C3 and IgG‐stained sections were visualized using an Olympus fluorescent microscope with digital camera (Olympus, Southend, UK) and images analysed using Image‐Pro Plus software (Media Cybernetics, Silver Spring, MD, USA). Ten glomeruli were examined per section, with mean fluorescence intensity expressed in arbitrary fluorescence units (AFU). Neutrophils were assessed based on polymorphic nuclei from PAS sections and counted in 50 glomeruli per section.

### Plasma C3 levels

Plasma C3 levels were measured using a sandwich enzyme‐linked immunosorbent assay (ELISA). NUNC Maxisorp 96‐well polystyrene plates were coated with polyclonal goat anti‐mouse C3 capture antibody (MP Biomedical, Cambridge, UK) and incubated overnight at 4°C. Plates were then washed and blocked with 2% bovine serum albumin (BSA) in washing buffer. After blocking, plates were washed again and plasma samples were added (1 : 12000 dilution) and incubated for 1 h at room temperature. Standards of mouse C3 were obtained using an acute phase serum (Calbiochem, Hertfordshire, UK). Following incubation, plates were washed and the detection antibody [horseradish peroxidase (HRP)‐conjugated form of capture antibody] (MP Biomedical) was added at a dilution of 1 : 25000 for 1 h at room temperature. Plates were developed using HRP substrate tetramethylbenzidine (TMB) substrate and the reaction was stopped with 20 µl stop solution, and the plates read at 450 and 540 nm.

### Plasma cytokine and chemokine levels

Plasma cytokine and chemokine levels were measured using Biolegend LEGENDplex (Biolegend, San Diego, CA, USA), according to the manufacturer's instructions.

### Statistical analysis

Parametric results were expressed as mean ± standard error (s.e.), while the median was used for non‐parametric data. Differences between two experimental groups were calculated using the unpaired *t*‐test for parametric data and Mann–Whitney *U*‐test for non‐parametric data. Comparison between multiple experimental groups was achieved using analysis of variance (anova) followed by Bonferroni's multiple comparison test if data were parametric, and the Kruskal–Wallis test followed by Dunn's multiple comparison test if data were non‐parametric. Comparison between experimental groups in the percentage of mice showing haematuria and proteinuria at each time‐point was performed using Fisher's exact test. Cut‐off for significance was set at *P* < 0·05.

## Results

### A hyper‐TGRL environment enhances renal damage during ANTN

**Figure 1:**
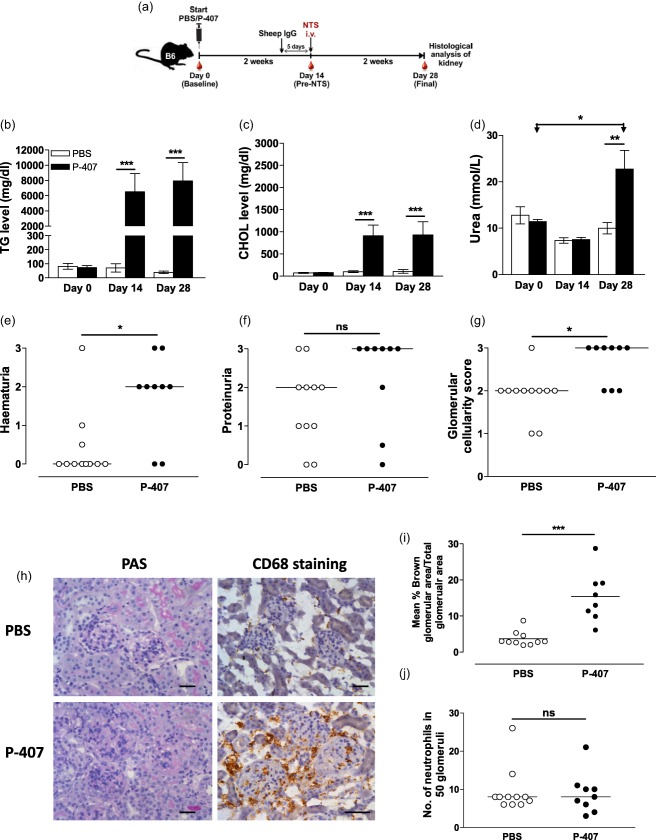
Poloxamer 407 (P‐407)‐induced hyper‐triglyceride‐rich lipoprotein (TGRL) state promotes kidney damage in the accelerated nephrotoxic nephritis (ANTN) model. (a) Schematic representation of the experimental design. A hyper‐TGRL state was induced in B6 mice by injecting 10 mg P‐407 intraperitoneally (i.p.) every other day for 28 days. Phosphate‐buffered saline (PBS)‐injected mice served as controls. Nine days post P‐407 treatment, the mice were immunized with sheep immunoglobulin (Ig)G followed 5 days later by intravenous (i.v.) injection of nephrotoxic serum (NTS). The mice were monitored for a further 2 weeks. Plasma levels of TG (b), cholesterol (CHOL) (c) and urea (d) at three time‐points, before commencing P‐407 injections (day 0), 14 days following P‐407 administration and prior to NTS injection (day 14) and 2 weeks after the induction of ANTN (day 28). Values represent mean ± standard error (s.e.); *n* = 9 in the P‐407 group, *n* = 11 in the PBS group; *P*‐values using the unpaired *t*‐test when comparing two groups and the paired *t*‐test when comparing days 0 and 28 within the same group. Due to insufficient blood collection a few samples were not available at days 0 and 14. Haaematuria (e) and proteinuria (f) measured by urine dipstick at day 28. (g) Glomerular cellularity score of periodic acid‐Schiff (PAS)‐stained kidney sections. (e–g) Horizontal bars indicate median; each circle represents a single mouse, Mann–Whitney *U*‐test. (h) Representative images of CD68‐ and PAS‐stained kidney sections at day 28 (scale bar = 50 μm). (i) Quantification of CD68 at day 28. Data are expressed as mean percentage of brown‐stained glomerular area/total glomerular area for 10 glomeruli per section. Each dot represents the mean % of 10 glomeruli per mouse, *n* = 8 in the P‐407 group, *n* = 10 in the PBS group. Horizontal bars indicate the mean, *P*‐value by unpaired *t*‐test. (j) Number of neutrophils counted in 50 glomeruli per section. Each dot represents one mouse. Horizontal lines denotes median, Mann–Whitney *U*‐test. *P < 0·05; **P < 0·01; ***P < 0·001; n.s. = not significant.

To define the effect(s) of high TGRL levels on the progression of renal disease and end‐organ damage we induced ANTN, a well‐established experimental model of immune complex (IC)‐mediated renal injury 22, in B6 mice that had been rendered hyperlipidaemic using P‐407. B6 mice, treated with PBS or P‐407, were pre‐sensitized with sheep IgG after 9 days and received NTS at day 14 (Fig. 1a). At this time‐point (day 14) all the animals exhibited very elevated levels of TG (6497 ± 848.6 mg/dl, mean ± s.e.) and a less pronounced increase in their CHOL levels (911·5 ± 85·27 mg/dl, mean ± s.e.) when compared to the PBS‐injected group (70·21 ± 8·77 mg/dl and 101·4 ± 5·91 mg/dl, mean ± s.e., respectively; *P* < 0·001, unpaired *t*‐test) (Fig. 1b,c). However, despite the changes in the lipid profile, the animals showed no evidence of renal impairment (normal urea levels and absence of haematuria and proteinuria on urinalysis) (Fig. 1d and data not shown). The P‐407 treatment, however, can trigger an accumulation of CD68^+^ cells in the kidneys, especially in the glomeruli 19.

Two weeks after the administration of NTS (day 28 of P‐407 treatment), haematuria and uraemia were increased significantly in the P‐407‐treated mice compared to controls, while proteinuria did not differ (Fig. 1d–f). However, the development of proteinuria occurred earlier in the P‐407‐injected mice, i.e. 2 days following NTS administration (Supporting information, Fig. S1). In the hyper‐TGRL mice there was enhanced glomerular cellularity (Fig. 1g), and immunohistochemical staining for CD68 revealed a significant glomerular accumulation of CD68^+^ cells (Fig. 1h,i), but not of neutrophils (Fig. 1j).

**Figure 2:**
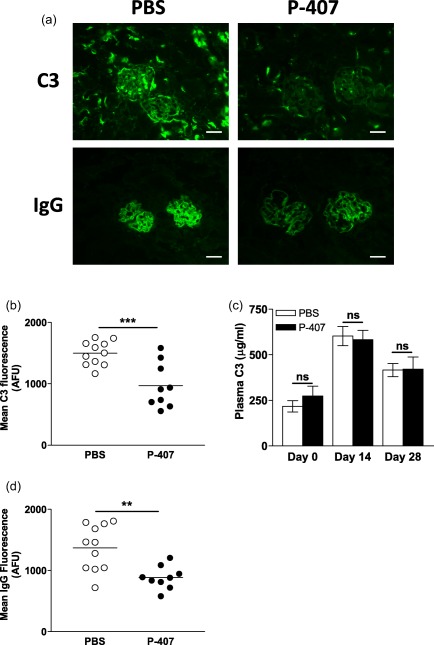
The hypersensitive response to accelerated nephrotoxic nephritis (ANTN) in hyper‐triglyceride‐rich lipoprotein (TGRL) mice does not appear to be complement‐mediated. (a) Representative images of glomerular C3 (top panels) and immunoglobulin (Ig)G (bottom panels) staining at day 28 in chow‐fed B6 mice treated with 10 mg poloxamer 407 (P‐407) or phosphate‐buffered saline (PBS), as shown in Fig. 1a. (scale bar = 50 μm). (b) Quantification of glomerular C3 expressed as arbitrary fluorescence units (AFU). Data shown are the mean fluorescent signal in 10 glomeruli per section. Each dot represents a mouse and horizontal bars denote the mean. *P*‐value calculated using the unpaired *t*‐test. (c) Plasma C3 levels (μg/ml) measured at the indicated time‐points. Data are presented as mean ± standard error (s.e.). *P*‐value by the unpaired *t*‐test. Due to insufficient blood collection a few samples were not available at days 0 and 14. (d) Quantification of glomerular IgG expressed as AFU. Each dot represents the mean fluorescent signal in 10 glomeruli per section from each mouse. Horizontal bars denote the mean, unpaired *t*‐test. **P < 0·01; ***P < 0·001; n.s. = not significant.

We next examined glomerular C3 deposition. Unexpectedly, glomerular C3 deposition was significantly lower in the P‐407‐treated mice when compared to the PBS‐injected controls (*P* < 0·001) (Fig. 2a,b). The difference in glomerular C3 deposition was not due to differences in circulating C3 levels, as these were comparable between the experimental groups throughout the course of the ANTN model (Fig. 2c). The reduced C3 staining was associated with markedly decreased glomerular IgG deposition in the P‐407‐treated mice compared to the controls (*P* < 0·01) (Fig. 2a,d). To explore whether the increased renal damage in the hyper‐TGRL mice was due to an abnormal innate immune response to NTS, we administered NTS to PBS‐ and P‐407‐treated mice without prior immunization with sheep IgG. Both groups showed enhanced proteinuria 24 h after the administration of NTS compared to their respective non‐NTS‐injected controls. However, the amount of proteinuria was not different between the hyper‐TGRL and the normolipidaemic mice (Supporting information, Fig. S2).

We then tested lower doses of P‐407 (5 mg and 2·5 mg), which resulted in lower levels of TG (3298 ± 157·2 and 965·7 ± 117·8 mg/dl, respectively, mean ± s.e.) and CHOL (395·3 ± 20·01 and 265 ± 15·35 mg/dl, mean ± s.e.) (Supporting information, Fig. S3a,b). At these lower levels of TG we found no increased glomerular damage (Supporting information, Fig. S3d–f), indicating that TG levels greater than ∼4000 mg/dl were required to aggravate the renal damage triggered by the ANTN model.

**Figure 3:**
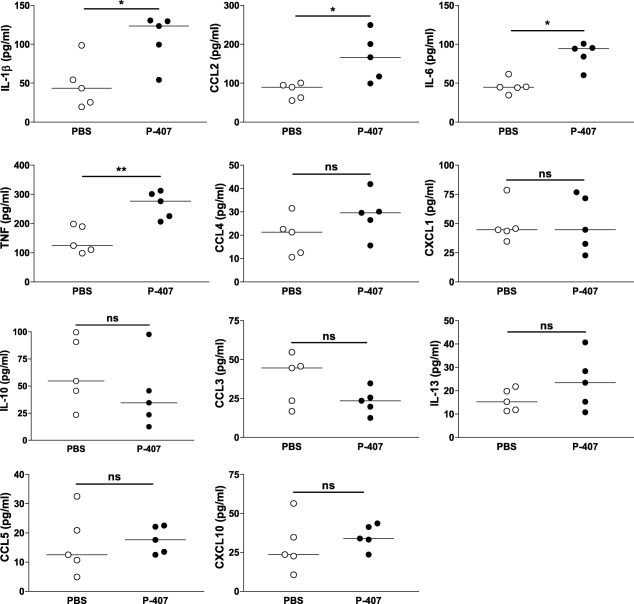
Cytokine and chemokine levels in hyper‐triglyceride‐rich lipoprotein (TGRL) mice following accelerated nephrotoxic nephritis (ANTN). Plasma levels measured at day 28 in chow‐fed B6 mice injected with either 10 mg poloxamer 407 (P‐407) or phosphate‐buffered saline (PBS), as Fig. 1a, *n* = 5 in each group. Each dot represents a mouse; horizontal bars indicate the median; Mann–Whitney *U*‐test. *P < 0·05; **P < 0·01; n.s. = not significant.

To investigate the mechanisms driving the hypersensitive response to ANTN seen with the P‐407‐treated mice, we measured a panel of cytokines and chemokines. As shown in Fig. 3, at day 28 the plasma levels of some inflammatory cytokines such as interleukin (IL)‐1β, IL‐6 and tumour necrosis factor (TNF)‐α were higher in P‐407‐treated animals compared to PBS‐injected controls at the end‐point. There was also an increase in CCL2, but not in other chemokines. Together these data demonstrate that a TGRL rich environment primes the kidney for enhanced injury in response to ANTN. This effect seems to be unrelated to IC deposition, complement activation or neutrophil infiltration, but is accompanied by an increased CD68^+^ cell infiltration and an enhanced inflammatory response.

### LDL‐driven hypercholesterolaemia does not promote renal injury following ANTN

**Figure 4:**
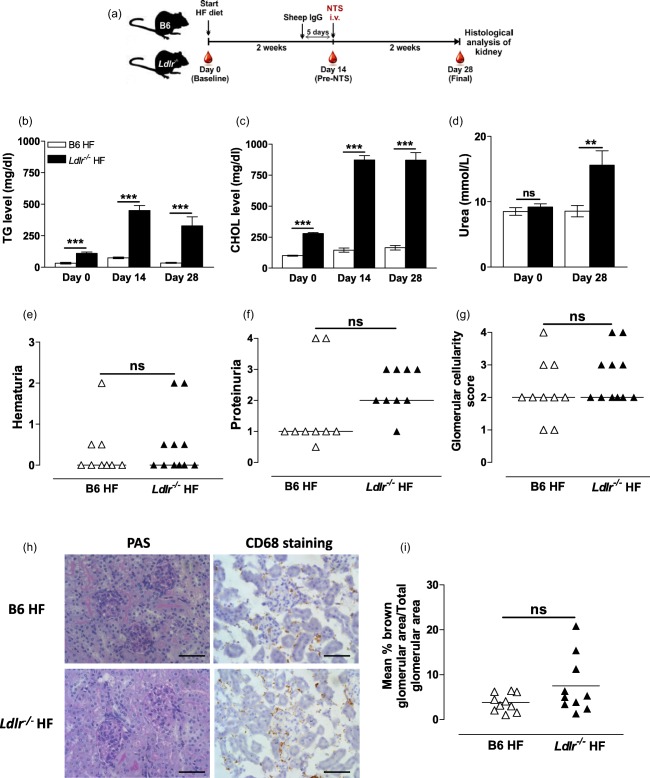
No enhancement of renal damage in diet‐induced hypercholesterolemic *Ldlr^–/–^* animals following accelerated nephrotoxic nephritis (ANTN). (a) Schematic representation of the experiment conducted in *Ldlr^–/–^* and B6 female mice (*n* = 7–11 in each group) fed a high‐fat (HF) diet. Nine days following diet modification, the mice were immunized with sheep immunoglobulin (Ig)G, followed 5 days later by intravenous (i.v.) injection of nephrotoxic serum (NTS). Blood was collected at baseline before commencement of HF diet (day 0), after 2 weeks of diet modification and before NTS injection (day 14), and 2 weeks following NTS injection (day 28). Plasma levels of triglycerides (TGs) (b), cholesterol (CHOL) (c) and urea (d) measured at the indicated time‐points. Urea levels were not available for one mouse in each group at day 0. (b–d) Data are presented as mean ± standard error (s.e.); *P*‐values calculated using the unpaired *t*‐test. Haematuria (e) and proteinuria (f) assessed by urine dipstick at day 28. Not all urine samples were available for the analysis. (g) Glomerular cellularity score (0–4) assessed on periodic acid‐Schiff (PAS)‐stained renal tissue in a blinded fashion. (e–g) Each symbol represents a mouse; horizontal bars represent median; *P*‐value calculated using the Mann–Whitney *U*‐test. (h) Representative images of PAS‐ and CD68‐stained renal tissue (scale bar = 50 μm). (i) Quantification of CD68 staining. Data expressed as mean percentage of brown‐stained glomerular area/total glomerular area for 20 glomeruli per section. Each dot represents the mean % of 20 glomeruli per mouse; horizontal bars indicate the mean; *P*‐value calculated using the unpaired *t*‐test. **P < 0·01, ***P < 0·001; n.s. = not significant.

**Figure 5:**
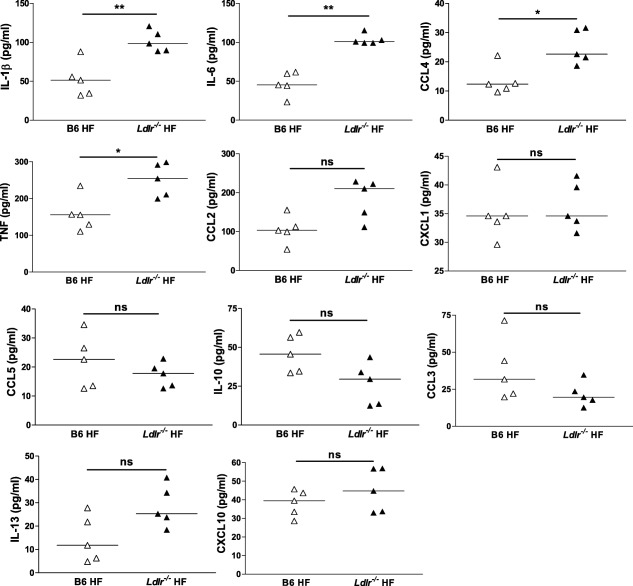
Cytokine and chemokine levels in high‐fat (HF)‐fed *Ldlr^–/–^* mice following accelerated nephrotoxic nephritis (ANTN). Plasma levels measured at day 28 in *Ldl^–/‐‐^* and B6 female fed HF diet. Each dot represents a mouse; horizontal bars indicate the median; Mann–Whitney *U*‐test; *n* = 5/group. *P < 0·05, **P < 0·01; n.s. = not significant.

The P‐407‐induced hyper‐TGRL state was accompanied by increased CHOL levels; thus, using this model we could not rule out a potential contribution of CHOL to the kidney insult. To explore the role of CHOL in this model we induced ANTN in a model of dyslipidaemia characterized by predominant hypercholesterolaemia : low‐density lipoprotein receptor‐deficient (*Ldlr^–/–^*) mice on a HF diet. *Ldlr^–/–^* and B6 mice were fed a HF diet for a period of 28 days, during which ANTN was induced (Fig. 4a). At baseline, the plasma levels of both CHOL and TG were generally higher in the *Ldlr^–/–^* animals than in the B6 mice prior to any dietary intervention (Fig. 4b,c). Fourteen days after the commencement of HF diet, the HF‐fed *Ldlr^–/–^* group showed significantly higher plasma levels of TG and CHOL (450·7 ± 39·43 mg/dl and 873·0 ± 34·03 mg/dl, respectively) compared to the HF‐fed B6 group (75·12 ± 7·028 mg/dl and 145·3 ± 16·46 mg/dl) (Fig. 4b,c). Notably, the hypercholesterolaemia achieved by diet modification in the *Ldlr^–/–^* mice was comparable to that observed using the 10 mg P‐407 dose (Fig. 1c), whereas the TG levels were much lower (Fig. 1b). At the end of the experiment the *Ldlr^–/–^* HF group had higher urea levels, but the uraemia was still within the normal range (below 20 mmol/l) (Fig. 4d). Haematuria, proteinuria, glomerular cellularity and glomerular CD68^+^ cell numbers did not differ between the *Ldlr^–/–^* HF and B6 HF mice (Fig. 4e–i). Of note, in the HF‐fed *Ldlr^–/–^* mice, as in the P‐407‐treated animals, we detected an enhanced cytokine response (Fig. 5). Thus, the *Ldlr^–/–^* HF mice, despite cholesterol levels comparable to the P‐407 treated animals, did not display a hypersensitive response to ANTN, suggesting a more detrimental role for hypertriglyceridaemia than hypercholesterolaemia in the renal injury.

## Discussion

Elevated TGRL levels are recognized as a risk factor for cardiovascular disease 13, 25, 26, while their contribution to renal disease remains poorly understood 27, 28. Here we show that a hyper‐TGRL state can be detrimental to the kidney. Animals with high TGRL levels displayed a hypersensitive response to renal injury triggered by the ANTN model with increased haematuria and glomerular cellularity. The increased renal damage in the hyper‐TGRL mice was accompanied by marked accumulation of CD68^+^ cells in the glomeruli implicating a role for these cells in mediating the injurious effects. By comparing animals with different TG levels but comparable CHOL levels, our data indicate that a hyper‐TGRL status is more detrimental to the kidneys than LDL‐driven hypercholesterolaemic conditions.

Dyslipidaemia and renal disease are commonly co‐encountered in clinical practice. Dyslipidaemia may occur as a complication of the primary renal disease itself, as seen in the nephrotic syndrome 1, or may precede its development as part of metabolic (e.g. MS and T2D) or inflammatory (e.g. SLE) conditions increasing the risk for renal complications 5, 10. The lipid profile seen in these conditions is dominated by hypertriglyceridaemia due to an accumulation of TGRLs. Several human studies have linked such a lipid profile to renal disease 29, 30, 31. However, establishing the precise role of TGRLs in renal disease has been hindered by other confounding factors. The P‐407 compound can induce a hyper‐TGRL state in the absence of hyperinsulinaemia and/or hyperglycaemia, the most common confounding factors complicating other hypertriglyceridaemic animal models 32. In addition, P‐407 treatment has been shown to have no hepatotoxicity 33, 34 nor to cause overt inflammation 19. Here we show that the P‐407‐induced hyper‐TGRL environment can prime the kidney for renal injury following a local insult. Although the detrimental effects of a hyper‐TGRL state were observed mainly under extremely high TGRL levels, our findings cannot exclude harmful effects by lower TGRL levels. In our study the mice were exposed to a hyper‐TGRL environment only for a short period (28 days), whereas patients with MS and/or T2D suffer from a hyper‐TGRL state for years. Thus one could speculate that a mild hyper‐TGRL environment could still be detrimental, but a longer period may be required for the consequences to be manifest.

Macrophages appear to play a role in mediating the enhanced inflammatory response to ANTN under hyper‐TGRL conditions, as their numbers were greatly increased in the glomeruli of P‐407‐treated mice compared to their normolipidaemic controls. We have reported migration of the ‘non‐classical’ Gr1^low^ monocytes into tissue with enhanced accumulation of CD68^+^ macrophages in different organs, including the kidneys, after 2‐week treatment with P‐407, a time‐point that corresponds to the time of NTS administration in the current study, but their presence was not associated with detectable renal injury 19. However, the data reported here using the ANTN model indicate that the tissue CD68^+^ macrophages might amplify the inflammatory response triggered by an insult. Therefore, what may have started as a beneficial physiological reaction aiming to maintain tissue homeostasis by clearing intrarenal lipids during hyper‐TGRL conditions may become detrimental once its physiological limits are exceeded, especially during inflammation.

A role for lipids in promoting renal damage has been reported. The lupus‐prone (NZB×W)F_1_ mice developed accelerated renal disease with higher proteinuria and increased mortality when fed a HF diet 7. Sprague–Dawley (SD) rats fed a high CHOL diet developed proteinuria 10 weeks after diet modifications, and this was also associated with enhanced macrophage accumulation 9. In both studies, the hyperlipidaemia was dominated by hypercholesterolaemia rather than hypertriglyceridaemia 7, 9. Here we show that not only hypercholesterolaemia, but also hypertriglyceridaemia and a hyper‐TGRL environment, are harmful to the kidneys. This is consistent with observations in the hypertriglyceridaemic obese Zucker rats that develop focal and segmental glomerulosclerosis with proteinuria and impaired creatinine clearance associated with renal macrophage accumulation as they age 35, 36. However, the obese Zucker rat model is also known to have insulin resistance with or without hyperglycaemia, both risk factors for renal disease 37. In contrast, the P‐407 model generates a hyper‐TGRL state in the absence of these confounding factors 32. This enabled us to separate the effects of TGRLs from those of insulin resistance and hyperglycaemia. Although the findings in the P‐407‐treated mice and in the obese Zucker rats are consistent, hypertriglyceridaemic Nagase analbuminaemic rats (NAR) did not display signs of renal injury and macrophages were not detected in their kidneys at 8 months of age 9. The discrepancy with our data may be because the increase in TGs in the NAR model is much lower than that achieved by P‐407 administration, which corroborates the notion of a threshold effect. Moreover, although the hypertriglyceridaemia seen in the NAR is secondary to VLDL accumulation, the lipid profile seen in NAR is also characterized by high HDL levels 38. As the HDL lipoprotein fraction is known to infer protection against atherosclerosis, we speculate that a similar protective effect may have occurred in the kidney in this model 39.

Feeding HF diet to *Ldlr^–/–^* mice results in hyperlipidaemia, driven largely by hypercholesterolaemia secondary to LDL accumulation with mild hypertriglyceridaemia. Despite the increased CHOL levels we could not detect any significant renal damage or accumulation of CD68^+^ macrophage in the kidneys of the HF *Ldlr^–/–^* mice, despite a comparable cytokine/chemokine profile with the P‐407‐treated mice. The similar systemic inflammatory response, however, does not exclude a different effect of TGRLs and CHOL on renal injury. It is known that some fatty acids, by‐products of TGRL degradation and cholesterol bind to different nuclear receptors, which can modulate distinct downstream effector functions 40. Thus, the divergence of the glomerular response in these two dyslipidaemic models may be due to different lipid metabolites or other signalling pathways. Our findings in the HF *Ldlr^–/–^* mice also differ from those reported in high CHOL diet‐fed SD rats where both renal damage and macrophage accumulation were seen 9. In addition to the difference in the duration of hyperlipidaemia between the two studies, the hypercholesterolaemia observed in the *Ldlr^–/–^* mice is secondary to LDL accumulation, whereas in the high CHOL‐fed SD rats it is due primarily to VLDL and IDL accumulation 9. The differences in the lipid profile, along with the duration of hypercholesterolaemia between the two models, may explain their contrasting renal phenotype.

In summary, our data demonstrate that a hyper‐TGRL environment primes the kidney to enhanced damage in the ANTN model. These observations may have important clinical implications. The P‐407‐induced hyper‐TGRL lipid profile recapitulates the profiles seen in a variety of metabolic and inflammatory conditions such as MS, T2D and SLE, where renal involvement is well documented. Moreover, as Zilversmit has long advocated a role for the postprandial hyper‐TGRL state in the development of atherosclerosis 13, our study indicates that this role may extend to inflammatory kidney conditions.

## Author contributions

M. F. S. performed the experiments, interpreted the data and wrote the paper; M. M. R., M. S. and K. J. W. performed some experiments; M. C. P. assisted with data interpretation and edited the paper; H. T. C analysed the histology; K. J. W and M. B. designed the research, analysed the data and edited the paper.

## Disclosure

The authors declare no financial or commercial conflicts of interest.

## Supporting information

Additional Supporting information may be found in the online version of this article at the publisher's web‐site:

**Fig. S1**. Haematuria and proteinuria in hyper‐triglyceride‐rich lipoprotein (TGRL) and normolipidaemic mice during the accelerated nephrotoxic nephritis (ANTN) model.
**Fig. S2**. Proteinuria 24 h after the administration of the nephrotoxic serum.
**Fig. S3**. ANTN model in mice with less pronounced high‐triglyceride‐rich lipoprotein (TGRL) conditions.
