# Supplementary material for: A triglyceride‐rich lipoprotein environment exacerbates renal injury in the accelerated nephrotoxic nephritis model
Source: Clin Exp Immunol. 2018 Mar 9;192(3):337–47. doi: 10.1111/cei.13111 (PMC5980512; doi:10.1111/cei.13111)
Supplement: Supplementary file 1 — Fig. S1. Haematuria and proteinuria in hyper‐triglyceride‐rich lipoprotein (TGRL) and normolipidaemic mice during the accelerated nephrotoxic nephritis (ANTN) model. Fig. S2. Proteinuria 24 h after the administration of the nephrotoxic serum. Fig. S3. ANTN model in mice with less pronounced high‐triglyceride‐rich lipoprotein (TGRL) conditions. [file CEI-192-337-s001.pptx]

## Slide 1
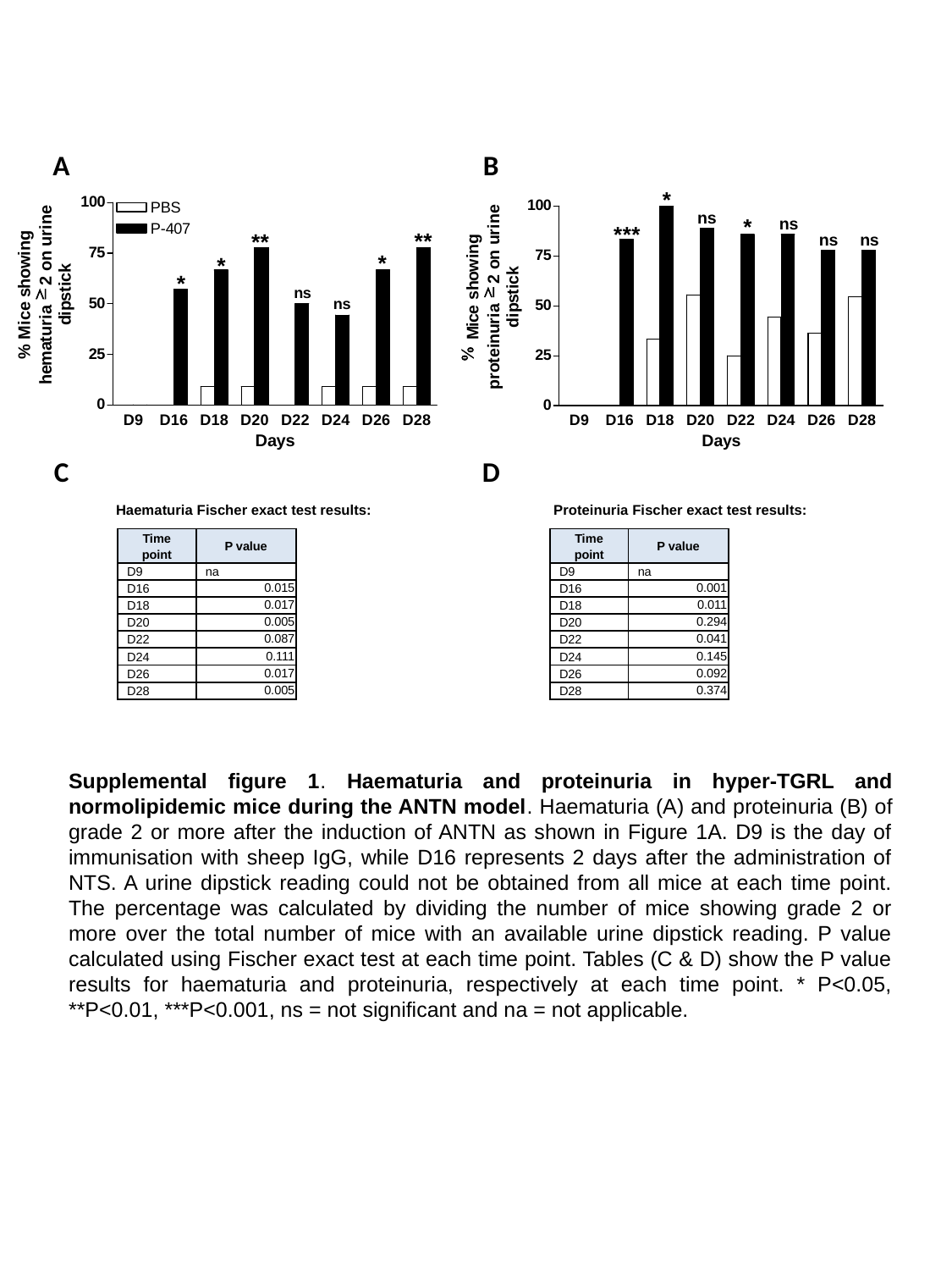

A
B
C
D
Haematuria Fischer exact test results:
Proteinuria Fischer exact test results:
| Time point | P value |
| --- | --- |
| D9 | na |
| D16 | 0.015 |
| D18 | 0.017 |
| D20 | 0.005 |
| D22 | 0.087 |
| D24 | 0.111 |
| D26 | 0.017 |
| D28 | 0.005 |
| Time point | P value |
| --- | --- |
| D9 | na |
| D16 | 0.001 |
| D18 | 0.011 |
| D20 | 0.294 |
| D22 | 0.041 |
| D24 | 0.145 |
| D26 | 0.092 |
| D28 | 0.374 |
Supplemental figure 1. Haematuria and proteinuria in hyper-TGRL and normolipidemic mice during the ANTN model. Haematuria (A) and proteinuria (B) of grade 2 or more after the induction of ANTN as shown in Figure 1A. D9 is the day of immunisation with sheep IgG, while D16 represents 2 days after the administration of NTS. A urine dipstick reading could not be obtained from all mice at each time point. The percentage was calculated by dividing the number of mice showing grade 2 or more over the total number of mice with an available urine dipstick reading. P value calculated using Fischer exact test at each time point. Tables (C & D) show the P value results for haematuria and proteinuria, respectively at each time point. * P<0.05, **P<0.01, ***P<0.001, ns = not significant and na = not applicable.

## Slide 2
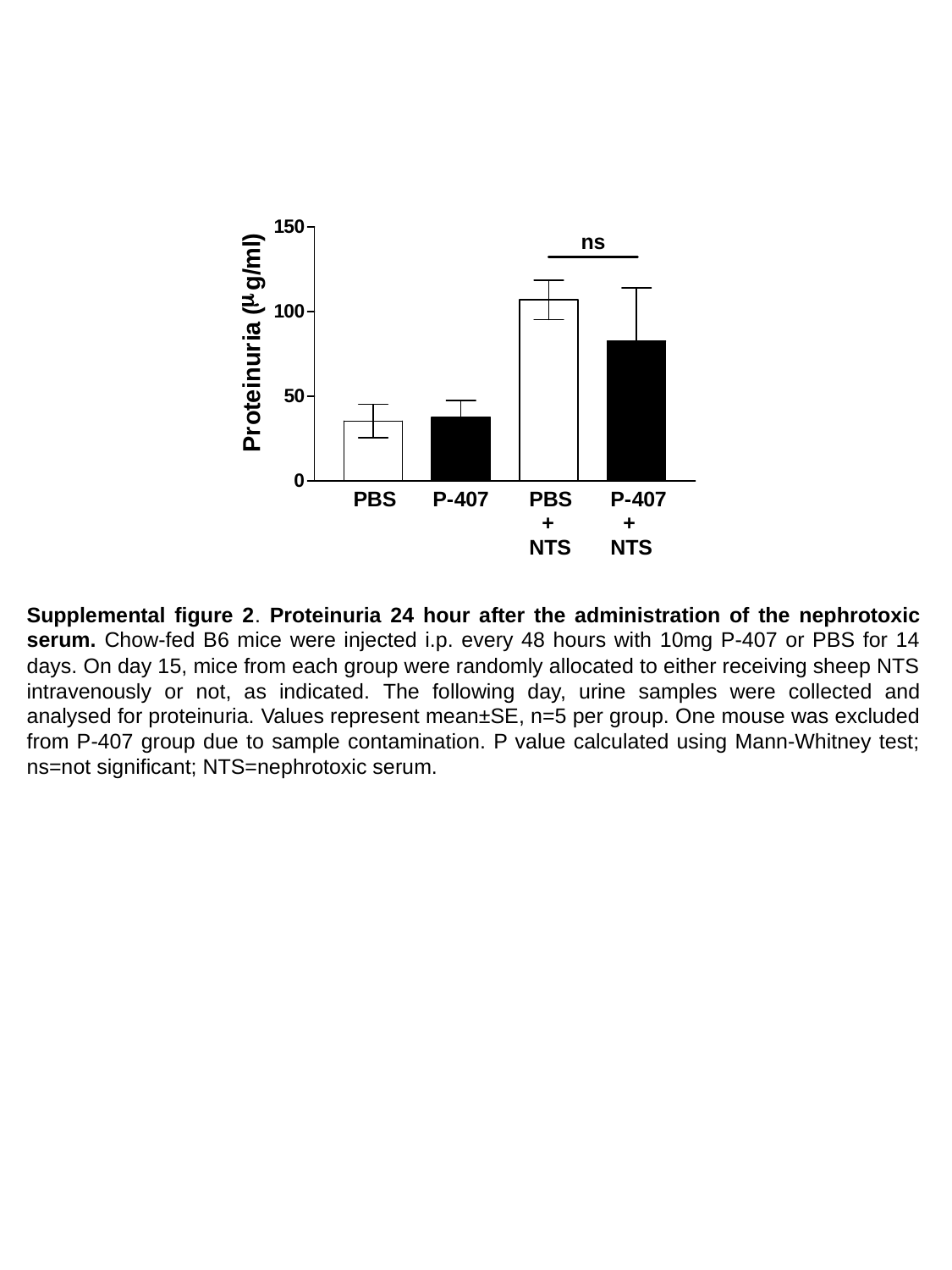

Supplemental figure 2. Proteinuria 24 hour after the administration of the nephrotoxic serum. Chow-fed B6 mice were injected i.p. every 48 hours with 10mg P-407 or PBS for 14 days. On day 15, mice from each group were randomly allocated to either receiving sheep NTS intravenously or not, as indicated. The following day, urine samples were collected and analysed for proteinuria. Values represent mean±SE, n=5 per group. One mouse was excluded from P-407 group due to sample contamination. P value calculated using Mann-Whitney test; ns=not significant; NTS=nephrotoxic serum.

## Slide 3
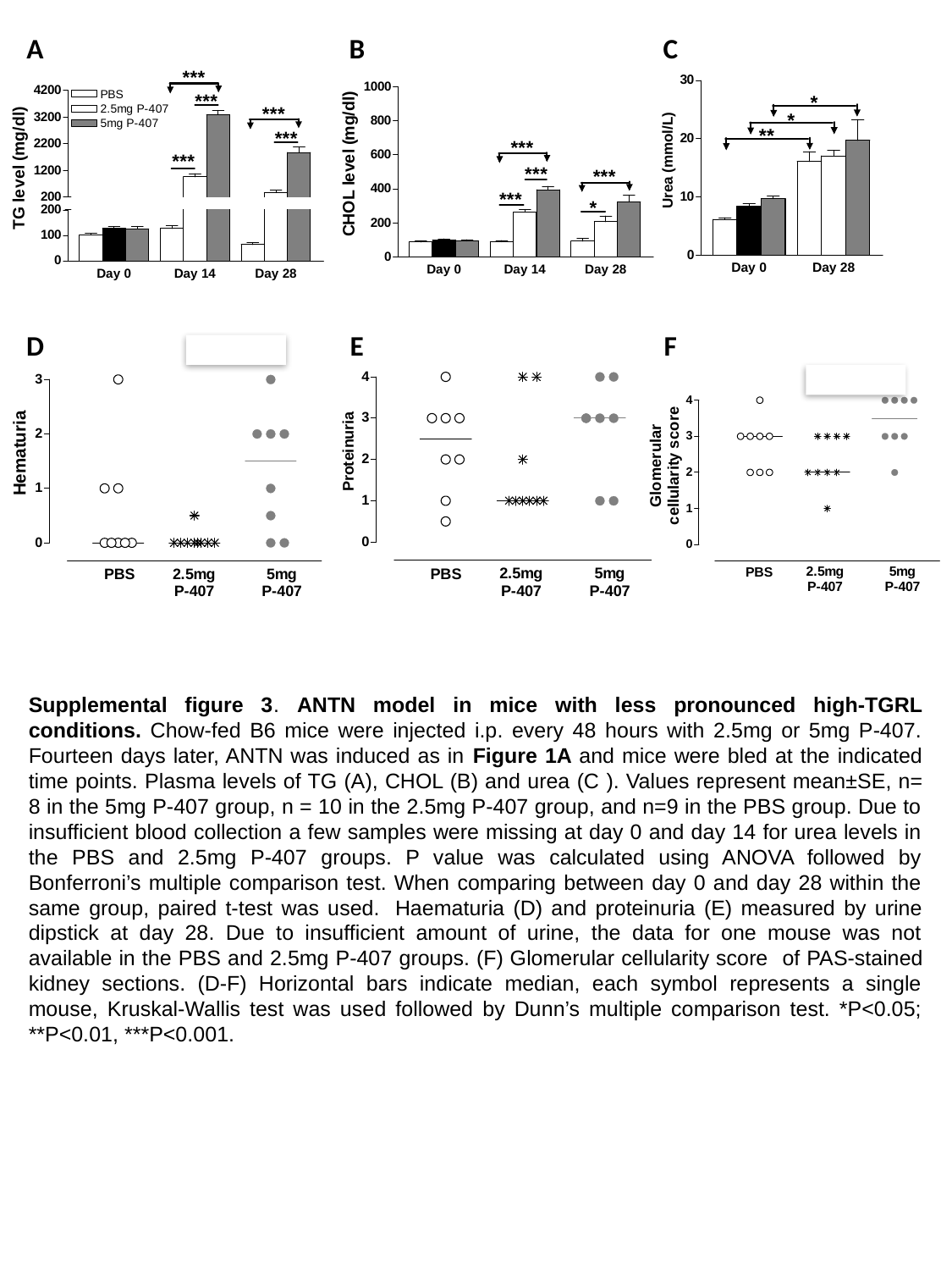

A
B
C
E
F
D
Supplemental figure 3. ANTN model in mice with less pronounced high-TGRL conditions. Chow-fed B6 mice were injected i.p. every 48 hours with 2.5mg or 5mg P-407. Fourteen days later, ANTN was induced as in Figure 1A and mice were bled at the indicated time points. Plasma levels of TG (A), CHOL (B) and urea (C ). Values represent mean±SE, n= 8 in the 5mg P-407 group, n = 10 in the 2.5mg P-407 group, and n=9 in the PBS group. Due to insufficient blood collection a few samples were missing at day 0 and day 14 for urea levels in the PBS and 2.5mg P-407 groups. P value was calculated using ANOVA followed by Bonferroni’s multiple comparison test. When comparing between day 0 and day 28 within the same group, paired t-test was used.  Haematuria (D) and proteinuria (E) measured by urine dipstick at day 28. Due to insufficient amount of urine, the data for one mouse was not available in the PBS and 2.5mg P-407 groups. (F) Glomerular cellularity score  of PAS-stained kidney sections. (D-F) Horizontal bars indicate median, each symbol represents a single mouse, Kruskal-Wallis test was used followed by Dunn’s multiple comparison test. *P<0.05; **P<0.01, ***P<0.001.
